# Near Infrared (NIR) Light Therapy of Eye Diseases: A Review

**DOI:** 10.7150/ijms.52980

**Published:** 2021-01-01

**Authors:** Qin Zhu, Shuyuan Xiao, Zhijuan Hua, Dongmei Yang, Min Hu, Ying-Ting Zhu, Hua Zhong

**Affiliations:** 1 Department of Ophthalmology, the First Affiliated Hospital of Kunming Medical University, Kunming 650031, China; 2 Department of Ophthalmology, the Second People's Hospital of Yunnan Province, Kunming 650021, China; 3 Tissue Tech Inc, Miami, Florida 33126, USA

**Keywords:** near infrared light, photobiomodulation therapy, mechanism, eye disease

## Abstract

Near infrared (NIR) light therapy, or photobiomodulation therapy (PBMT), has gained persistent worldwide attention in recent years as a new novel scientific approach for therapeutic applications in ophthalmology. This ongoing therapeutic adoption of NIR therapy is largely propelled by significant advances in the fields of photobiology and bioenergetics, such as the discovery of photoneuromodulation by cytochrome c oxidase and the elucidation of therapeutic biochemical processes. Upon transcranial delivery, NIR light has been shown to significantly increase cytochrome oxidase and superoxide dismutase activities which suggests its role in inducing metabolic and antioxidant beneficial effects. Furthermore, NIR light may also boost cerebral blood flow and cognitive functions in humans without adverse effects. In this review, we highlight the value of NIR therapy as a novel paradigm for treatment of visual and neurological conditions, and provide scientific evidence to support the use of NIR therapy with emphasis on molecular and cellular mechanisms in eye diseases.

## Introduction

Light is a form electromagnetic radiation characterized by particle- and wave-like properties. Electromagnetic radiation waves have unidirectional vectors, characterized by wavelengths (λ, distance in successive peaks), frequencies (oscillations per second), and amplitudes (difference of trough and peak). The energy particles within electromagnetic radiation includes photons, which travel at 3×10^8^ m per second. Therefore, a mixture of waves will have photons traveling at various amplitudes and frequencies that are scattered and absorbed, which can be reflected by numerous objects, such as biological materials. As more and more studies have been performed to determine on how to harness this energy, the applications of light to medical therapy has advanced dramatically worldwide, for example, laser therapy is now a common treatment in certain specialties and has been shown effective for many chronic diseases without side effects.

More recently, low-level light therapy in the far-red (FR) to near-infrared (NIR) range of the spectrum (~600-1000 nm), collectively termed photobiomodulation (PBM), has gained worldwide attention in recent years as a novel tool for experimental therapeutic applications in a variety of medical conditions. For instance, applications of PBM to medical disorders, has been shown to restore functions of damaged mitochondria by upregulating cytoprotective factors and protecting against damage 1. These findings suggest laser therapy may inhibit inflammation and promote immune response and wound healing for various tissues (reviewed in 2).

This review will concisely describe the history of the modality, the current proposed mechanisms of action of NIR light at the molecular, cellular, and nervous tissue levels, the clinical indications and applications of photobiomodulation therapy (PBMT), the results of clinical studies, and the potential other benefits of PBMT in eye diseases.

## NIR Light, LEDs, PBMT and Lasers

The light brightness and color are determined by the number of photons and energy of each photon. NIR light therapy uses directional low-power but high-fluency light, monochromatic or quasimonochromatic, from lasers or light-emitting diodes (LEDs) in the wavelengths, red to near-infrared, to mediate biological functions or to promote therapeutic effects in a safe way 3, 4 (also reviewed in 5).

A laser is defined as a device to emit light by optical amplification, theoretically contingent on the stimulation of electromagnetic radiation emission. The word “laser” originated from the acronym of Light Amplification through Stimulation of Emission Radiation. The first laser was invented by Theodore H. Maiman in Hughes Research Laboratories in 1960. After its development, the laser has been increasingly used as a medical therapy, including low light therapy, low laser therapy and phototherapy or PBMT.

PBMT is a kind of light therapy using non-ionizing light sources, such as broadband light, lasers, and LEDs in visible or infrared spectrum. PBMT uses low-power but effective monochromatic light from LEDs in the wavelengths from red to near-infrared region to mediate biological events or therapeutic effects without any side effects 3, 4. This has been shown to protect against retinal degeneration by reducing light-induced stress, inflammation and cell death 6, to promote contrast sensitivity and visual acuity in macular degeneration 7, to elevate phagocytosis, to reduce reactive oxygen species production 8, and to decrease structural and functional damages in Diabetic Retinopathy 9.

NIR and LEDs light can activate photoreceptor cytochrome *c* oxidase, leading to elevated energy metabolism, increased wound healing, and inhibited toxicity (reviewed in 10). The applications of NIR light is to convert luminous energy into metabolic energy to modify biological functions of cells. Compared to the effects of high-energy photon by lasers, which result in heating and destruction to living tissues and cells, NIR light is in the low level since the energy level of electromagnetic radiation is negatively associated to its wavelength. In addition, the tissue is usually targeted by low irradiances, which does not cause side effects because energy delivered from NIR light is low enough not to cause heating and tissue destruction but high enough to mediate cell and tissue functions. For example, photodynamic therapy has been used for skin condition applications without side effects 11, and electrical photoneuromodulation in neurons may be obtained without thermal effects 12. Clinically, the therapeutic optical window corresponding to the red to near-infrared wavelengths, matches the luminous energy which promotes excitation of susceptible intracellular molecules in vivo 13. That is why NIR light is termed near-infrared light therapy, based on the theory that some molecules in living cells and tissues may absorb photons, initiating signaling pathways triggered by light 14. This results in energy conversion in which certain molecules may be excited by light, reaching an electronically excited state, which changes their conformation and function and activates signaling pathways to mediate cellular metabolism. In contrast to conventional laser high energy, destructive effects such as enormous heating, tissue destruction by ablation, coagulation, dissection and vaporization does not occur.

## NIR Light Resources

On earth, the primary source of electromagnetic radiation is solar energy from sunlight. Solar energy contains multiple waves in the electromagnetic spectrum, for example, wavelengths in the visible spectrum is multidirectional and not synchronized in time and space. In contrast, NIR light is monochromatic, which allows high specificity in molecular biomodulation. NIR light may be obtained by LEDs arrays and lasers, which are applied to PBM of eyes. Lasers can produce efficient coherent light energy to permit high penetration of tissues and such constant beam delivers energy to circumscribed areas. The beam width from lasers may be enhanced by their coupling into fiber optic to allow distribution of energy to larger areas. LEDs can produce efficient but not coherent light in wavelengths between 4-10 nm. Nevertheless, LEDs only produce negligible heat, impossible for thermal injury 15. In addition, LEDs can be coupled to arrays with ergonomic functions, to allow delivery of efficient energy to a large area, for example, the brain. In fact, LEDs have been evaluated in human trials and have been approved by the FDA 16.

## NIR Photoreceptors and Targets

As NIR light can cause various changes in physiological and pathological conditions, it has long been suspected that the light must act on certain receptors in living cells and tissues. For example, opsins in animals absorbs light, which induces phototransduction within photoreceptors. Photoreceptors include specialized and nonspecialized photoreceptors in mammalian cells. Hemoglobin, melanin, oxyhemoglobin and water absorb light from broad ranges of wavelengths. In the wavelength range of 600-1000 nm, the absorption curve is minimal, supplying a window for light to activate certain photoreceptors. In fact, the cells are sensitive to light from far-red (FR) to NIR, which can promote photon therapy by change of cell metabolism, proliferation and migration. For instance, after 3 days under 628 nm light, mitochondrial respiratory and antioxidant genes are upregulated and apoptotic and stress-related genes are downregulated in human fibroblasts due to activation of cytochrome c oxidase 17. The question is what molecule(s) in cells was activated for the effective treatment by NIR light. It is inferred that the wavelengths in red and near-infrared range are responsible for effective photobiological therapy. Another example is photoreceptors in hemoglobin of mammalian tissues, showing differential light absorption according to redox state, which allows its quantification and oxygenation in clinical applications. Except hemoglobin, other photoreceptors in red and near-infrared range are heme-containing metalloproteins, for example, cytochrome c oxidase and myoglobin. Cytochrome c oxidase, which can be activated by light from FR to NIR range, cause cellular changes 16, 18. In addition, other molecules such as catalase, cryptochromes superoxide dismutase, cytochrome c, cytochrome b, guanylate cyclase and nitric oxide synthase also have photoreceptors 15.

Although many photoreceptors have been discovered, the mechanism of NIR light is still unclear because many molecules have photoreceptor capabilities. In addition, reaction by a molecular target to a specific effective wavelength may be multiple. For instance, flavoprotein and NADH-dehydrogenase have photoreceptors not only in the violet-to-blue but also in red and near-infrared spectral regions 18. In addition, both terminal oxidases and superoxide dismutase have absorption peaks at high wavelengths of 670-680 nm, overlapping in the absorption spectrum of different photoreceptors 18, 19. This implies that the effect of other photoreceptors to one biological effect cannot be excluded. Interestingly, research on the nervous system is the first to identify an important photoreceptor mediating biological effects by NIR light. Isolated mitochondria are responsive to irradiation by monochromatic light in red to near-infrared spectrum. Light could promote mitochondrial membrane potential, promote ATP exchange, RNA and protein synthesis by mitochondria, and increase usage of oxygen 20. The effects from NIR light on mitochondria are wavelength-specific, and molecules absorbing NIR light in living cells are likely respiratory components 21. Further research on mitochondrial photoreceptors has been performed by action spectra analysis-descriptions of biological responses to NIR light as function from a wavelength. For example, RNA synthesis in HeLa cells could be promoted by certain wavelengths, but not by other wavelengths. General speaking, the wavelength inducing maximal effect can be found in a range of effective wavelengths, which is helpful to identify photoreceptor-mediated signaling by NIR light. In fact, the bands in the action spectrum is in the absorption spectrum of mitochondrial cytochrome oxidase 18. Cytochrome oxidase is identified as a primary photoreceptor of light in red and near-infrared region in electromagnetic spectrum 22, 23, which is a key oxidase in cell bioenergetics in nerve cells from retina and brain 24. Cytochrome oxidase is the key enzyme in electron transfer 360 in mitochondria, catalyzing electron transfer to oxygen from cytochrome c, reducing ~95% of oxygen taken by eukaryotic cells. Cytochrome oxidase also acts as a redox-coupled proton pump to promote formation of transmembrane electrochemical gradient, and as a rate-limiting step to synthesize energy-storing ATP 25. Cytochrome oxidase is involved in neuronal and free radical metabolism, apoptosis process and glutamatergic regulation 26, 27. Cytochrome oxidase activity is strongly associated with neuronal function and is a specific marker for intraneuronal metabolic activity 27. In addition, cytochrome oxidase may only be the primary photoreceptor in its intermediate but not final forms by analysis of sequential irradiation 28, 29. Absorption spectra in cytochrome oxidase with different oxidative states are parallel to action spectra in biological responses of NIR light 28.

## NIR Light Effects and Applications

NIR light dosimetry is hormetic dose-response with a curve in a bell-shape or U-shape or biphasic shape, characterized by promotion of biological process at a low dose but inhibition of biological process at a high dose. Hormetic curves are better than linear curves in accurately predicting biological responses under pharmacological threshold 30. It is very important since stimulatory responses from NIR light are mild, ~ 30% to 60% above controls. In contrast, traditional dose-responses are usually increase by several folds. The hormatic dose-response is well known in various NIR light applications, because photo--stimulatory or photo-inhibitory effects are shown in low (0.001 J/cm^2^) or high (.10 J/cm^2^) energy densities respectively 31. Positive responses can be achieved in the dose range for desired therapeutic outcomes 16, 32, 33.

Examples of NIR light effects and applications are listed for our understanding of usefulness of NIR light for potential medical applications. For instance, wound healing could appear in rat skin under a ruby laser with low energy 34 or under Gallium laser producing 685- or 830-nm light 35. Spinal cord injury could be improved under 810-nm light in rat 36. NIR light was beneficial for treating gingival incisions 37, oral mucositis 38, ulcers of skin 39, and wound healing 40, nerve repair 41-43, in carpal tunnel syndrome 44, 45 and soft tissue injuries 46. Inflammation could appear in light-stressed retina 47-49. Anti-inflammation by microglial inhibitor naloxone could decrease photoreceptor degeneration in retina 50, 51. LED pretreatment may protect neurons from cyanide-induced apoptosis by inhibiting reactive oxygen synthesis and apoptosis and promoting energy metabolism 52. Recently, 670 nm red light has been found has potential for potential wild medical applications evidently by many research articles. For example, 670 nm light could protect neuronal cells under treatment of cyanide 16, protect photoreceptors in rat and promote wound healing in primate retina 53, increase mitochondrial metabolism, decrease retinal inflammation, and reduce oxidative cell stress, probably by respiratory chain complex I, II and IV to target cytochrome c oxidase, causing improved energy metabolism in mitochondria 54. 670 nm red light is a powerful neuroprotectant against light induced damage 6 and toxins 55. Treatment with 670 nm red light decreases retinal inflammation by increasing mitochondria membrane potential 56, improves retinal healing 15, such as reducing raised intracellular pressure in rat retina 57, retards aging retinal functions 58. 670 nm LED can mediate inflammation and innate immunity in neural retina in a mouse model of macular degeneration 59, probably by upregulating expression of cytochrome c oxidase, along with reduced inflammation 60. The respiration of aged retinal mitochondria can be improved by 670 nm light as demonstrated by increased mitochondrial performance and reduced inflammation via improved oxidation by activated cytochrome c oxidase. Aged retina can have a progressive increase of oxidation by 670 nm light within only 5 minutes 61. 670 nm light may also alter oxygen-mediated degeneration in retina in a mouse model, showing that 670 nm light decreases expression of oxidative stress markers and reduces in hyperoxia conditions 62. Pretreatment of 670 nm light can decrease lipid peroxidation and reduce complement propagation in retina degeneration 63. Low levels of 670 nm light may prevent against retinopathy by oxygen induction and lung damage by excessive oxygen 64 and modulate expression of genes involved with inflammation, oxidative metabolism and apoptosis 65. Although the precise mechanism is unknown, there is strong evidence to suggest that cytochrome c oxidase acts as the primary photo-receptors 66, boosting oxidative metabolism 67 and ATP production 18, and is probably linked to increase of cytochrome c oxidase and reduction of acrolein expression 68, driving reparative and protective mechanisms. In fact, abundant research results supports its potential benefits in retinal disease 33, stroke 69, 70, neurodegeneration 5, neuromuscular disorders 71, hair regrowth 72, memory 73 and mood disorders 74.

## Possible Mechanisms in NIR Light Therapy

Cytochrome oxidase is the primary NIR light photoreceptor, which is especially beneficial to eye and brain because the enzyme is the key enzyme in oxidative metabolism. Eyes and neurons rely on cytochrome oxidase for their metabolism. The effects of NIR light can be primary (during light exposure) and secondary (post light exposure). Primary effects are those of immediate photochemical changes in the photoreceptors activated by light. Therefore, those effects are dependent on light. For example, redox changes of the respiratory chain can be induced by NIR light, which promotes oxidation by cytochrome oxidase, linked to the bell-shaped and dose-dependent cellular responses 4. NIR light can also promote oxidation of cytochrome c by cytochrome oxidase, inducing elevated usage of oxygen, increased sensitivity of mitochondrial membrane and pore permeability in mitochondria 18, 19. Those effects are caused by increased electron flow in the mitochondrial electron transporting chain. In addition, NIR light can generate free radicals, through singlet oxygen by photodynamic action and superoxide ion by electron auto-oxidation. In addition, NIR light may produce transient heat in chromophore by electric or light oscillations 18. Such oscillations may affect various molecules in target tissues. NIR light may also strengthen hydrogen promoting energy transfer. Secondary effects from NIR light may appear as results of primary effects, for instance, biochemical and biological reactions which affects cellular homeostasis 4, 75, 76. NIR light may initiate various signaling pathways from mitochondria to nucleus by sending signals from mitochondria in which photoreceptors are activated, translocated to the nucleus, and in which gene expression is altered. In details, NIR light promotes beneficial changes of NAD/NADH ratio in mitochondria to induce release of nitric oxide by activated cytochrome oxidase, and thus to mediate ATP level. ATP can then activate its P2 receptors to cascade inward calcium currents, promoting release of calcium from its intracellular storages 77. Such changes of ATP can regulate level of cAMP, causing activation of various kinases to alter gene expression, which may regulate inflammation and wound healing.

PBM, or low light therapy, has been recognized for approximately 50 years. However, its cellular and molecular mechanisms are still not very clear. Recently, research on importance of cytochrome c oxidase in PBM has gained interactional attentions. The first hypothesis is that by stimulation of low light, the photons promote dissociation of inhibitory nitric oxide from this enzyme, promoting electron transport and ATP production, potentiating mitochondrial membrane and ATP production, and expression of transcription factors, which promote gene expression, protein synthesis, various cellular and tissue functions (reviewed in 13). PBM, by photon irradiation from NIR light with low energy, has a history of clinical application for treatment of soft tissue injuries and wound healing for decades. NIR light can penetrate tissues such as the brain, heart and spinal cord 78, 79, accelerate healing from ischemic heart injury 78, inhibit degeneration in injured optic nerve and retina 3, 78, and promote recovery in stroke 79-81. These effects by NIR light are clearly associated with the absorption spectrum of cytochrome oxidase 15. NIR light initiates changes of redox in cytochrome oxidase, causing activation of intracellular signaling to positively mediate mitochondrial function and intracellular protection by overexpression of cytoprotective factors 3, 16, 52, 79, 82-85. NIR light can also promote expression and accumulation of antioxidants and cytoprotective factors 55. For example, gene profile analysis show that NIR light promote noncoding RNAs (ncRNA) 65. To understand the role of the noncoding sequence is an interesting topic for further investigation.

PBM can be applied to clinic therapy to ease inflammation and pain, wound healing in bones and tendons via mitochondrial cytochrome c oxidase to improve electron transport, light ion channels (reviewed in 86). During PBM, cytochrome oxidase, a primary photoreceptor of NIR light, plays a critical role in a constructive role of recoveries of injured eye and brain tissues because the enzyme in mitochondria is critical for oxidation. For example, NIR light has robust neuroprotective effects in improvement and functional restoration of damaged retina by increase of mitochondrial function, improvement of blood flow to damaged neurons, increase of cell survival factors 87.

PBMT has become increasingly popular in human medicine in recent years. Recent investigations have demonstrated that laser therapy may regulate immune and inflammatory responses and promote wound healing in certain tissues 88. In addition, energy from PBMT are very limited without any concerns of heating and tissue destruction, but abundant for mediating cell functions. For example, PBMT can be used for photodynamic therapy of skin diseases 11 and for thermal therapy for neuronal diseases 12. Although cells are sensitive to certain wavelengths in electromagnetic spectrum in vitro, beneficial observations to wavelength are preferentially only a narrower range of wavelength in vivo. For instance, light in visible range (usually 400-700 nm) can penetrate and activate retinal cells with specific photopigments in cones and rods. The wavelength from red to near-infrared range is the best to cause beneficial effects in the cells without specific photopigments. This is probably due to the penetrating capacity of wavelengths in certain ranges to penetrate tissue. The lower the wavelengths are, for example, violet and ultraviolet wavelength, the less the wavelength to penetrate, whereas the wavelength in infrared red have higher penetration ability. In addition, energy at wavelengths < 600 nm is usually widely distributed in living tissues in vivo 13. For medical purposes, this suggests there is in vivo therapeutic optical ranges from red and near-infrared wavelengths. This optical window also corresponds to the capability of luminous energy to activate intracellular biological molecules 13. Interestingly, redshifted channel rhodopsin injected into blind rd1 mice may restore light responses of the mice from their retina and cortices to low orange light and ganglion cells from human retina 89. Therefore, NIR light is also termed near-infrared light therapy. That is, NIR light is hinged on the concept that living biological molecules can absorb photons, activating signaling pathways triggered by light 14. This biological process is called energy conversion based on the theory that biological molecules activated by light can reach electronically excited state from basal line, which results in changes of conformation and functions, thus inducing activation of certain signaling pathways to mediate cellular metabolism and recovery. Although tremendous progress has been made, details of mechanistic studies are required for further advance of NIR light applications to medical field.

## Potential Retinal Therapeutic Applications by NIR Light

Evidence has suggested that NIR light may restore biological function of damaged mitochondria, upregulate cytoprotective factors and inhibit apoptosis. Photons from NIR light can penetrate diseased retina, be absorbed by mitochondrial photoreceptors such as cytochrome c oxidase to promote mitochondrial energy beneficial metabolism, elevate production of cytoprotective factors and prevent apoptosis.

The examples of NIR light to potential medical applications are listed below. NIR light can protect retina in injury in which mitochondrial energy metabolism is damaged by a retinal injury model manipulated by methanol 53. NIR light may decrease damage in the layer outside nucleus and protect the wave amplitude of electroretinogram b 90. Surprisingly, protective effects from NIR light lasts as much as a month from damage, linked to increase of mRNA expression in neuroprotection. NIR may also protect photoreceptors, avoid cell death due to phototoxicity with decreased multiple gene expression induced by light damage 65. Remarkably, NIR light may reduce death of photoreceptors by 70% 91. NIR light can also inhibit cell degeneration of retinal ganglion cells caused by inhibitor rotenone to the mitochondrial complex I 1. NIR light treatment can also promote drusen, yellow deposits from macular degeneration due to aging, lower intraocular pressure as long as several months 6, indicating that NIR light can have beneficiaries in retinal and optic nerves.

Mitochondrial degeneration and oxidative damage of retina are shown in retinal degeneration and other damages, for example, aging macular degeneration, light-stimulated retinal damage, methanol intoxication and retinitis pigmentosa 92-94. NIR light may activate mitochondria signaling pathways to promote mitochondrial function, induce expression and activation of cytoprotective factors, inhibit oxidative stress, and protect neurons from death in in vitro neurons and in vivo animal models for neuronal injuries 5, 15, 16, 18, 76.

## Potential Optic Therapeutic Applications by NIR Light

Light penetration from NIR light rely on target tissues, wavelengths, and sources of NIR light. NIR light is safe but effective. Within near-infrared wavelengths, light can penetrate eyes at maximal level, but absorption of light by cornea and lens is at minimal level (approximately 10%) 95. The neurons from retina require high energy, or mitochondrial ATP to satisfy tissue requirements 96-98. Like other neurons, retinal neurons are very sensitive to depletion of glucose or lack of energy, oxidative stress and malfunction of mitochondria 99, 100. Mitochondrial malfunction plays a critical role in retinal neurodegeneration 101. Optic neuron damages are characterized by high morbidity and mortality, without any effective treatments at present 102-105. Alzheimer's patients have fewer retinal ganglion cells, ganglion axons, than healthy people 104, 106, 107. Leber's optic neuropathy, a mitochondrial disease, can lead to ~2% of all blindness patients in this world 108. Therefore, cellular degeneration from retinal ganglion cells is a major health problem in this world. Other genetic disorders include Leigh syndrome 109, 110, Friedreich's ataxia 111, myoclonic epilepsy due to ragged-red-fibers 112, mitochondrial acidosis and strokes 113, spastic paraplegia 114, and optic atrophy syndrome 115. Therefore, NIR light is significant for applications to eye and neuron therapies to retinal damage by correction of mitochondrial disorders. For instance, the beneficial NIR light can reverse optic nerve injuries by use of helium-neon laser in models of rabbit and rat 116, 117. In all, NIR light is effective on moderate certain injuries of eyes and nerves.

## Treatment of Myopia by NIR Light

As suggested, the myopia epidemic effects the entire world 118-124 (also reviewed in 125). Higher Myopia can result in severe retinal tears and detachment, choroidal degeneration, glaucoma and cataract, causing a large number of blindness in U.S. 126-130. Higher order aberrations refer to optical imperfections in eyes that affect quality of retinal image, resulting in low quality of retinal image. This principle has been applied to control of myopia by orthokeratology, near addition spectacle lenses and soft contact lenses with dual-focus and multi-focus, and its potential significance to myopia control is enormous 131. We have reported that 1% atropine retards progression of moderate myopia in Chinese school children in a mega clinical investigation 132. Therefore, such level of prevalence for myopia is a very serious problem in public health. As people around the world are aging, myopia prevalence is continually rising 133 unless we can devise ways to block, or at least reduce, myopia development in children.

Myopia, a refractive errors disease, is increasingly common, causing blindness worldwide, likely by light triggered retina-to-sclera changes 134. In postnatal refractive development, long-wavelength light may cause similar refractive error to that in myopia 135. However, long wavelengths are not closely associated with promotion of myopia development in an infant monkey model 136. Animals and human infants have eyes with large refractive errors 137-142. Orthokeratology, using gas permeable contact lenses to reduce refractive errors from myopia, is currently widely used for adolescent myopia control (reviewed in 143). Once myopia occurs, orthokeratology can retard myopia progression, by using prismatic bifocal lenses and multifocal contact lenses as soon as possible 13. Increased outdoor activities are also effective to prevent myopia occurrence and to retard shift of refractive error from myopia, but not to decrease progression of already myopic eyes (reviewed in 144). Through aging, the axial length is adjusted to match the eye's optics, reaching the state called emmetropia where retina is at the focal plane. The activation mechanism of emmetropization is believed that in the postnatal eye, elongation rate of eyes is set to align retina to eyes' focal plane 145-149. Emmetropization may respond to defocus by wavelength differently because animals may adjust refractive state by monochromatic light rather than white light, are relatively more hyperopic by blue light and relatively more myopic by red light 150. The composition of wavelength is closely associated with refractive errors and thus myopia, for example, flickering blue light promotes refractive decreases such as myopia, and upon returning to colony lighting, refractions may return to emmetropia, suggesting that flicker light may be closely linked to signaling defocus 151. Red light may alter refractive development by decreasing elongation of vitreous chamber and increasing choroidal thickness, suggesting that long-wavelength light with narrow band may retard axial elongation induced by hyperopic defocus and form deprivation, probably due to activating signals linked to myopic defocus 131. By constant adjustment of refractive state, emmetropization keeps approximate emmetropia in the entire postnatal period 146, 147, 152-156. In early postnatal period, emmetropization responds roughly to the refractive error. As people are maturing, optics at eye front gradually adjusts the focal plane to retina, retarding axial elongation and compensating for plus lens to make eyes emmetropic if wearing lens and hyperopic if lens are removed 146, 148, 152, 154, 157-164. In postnatal development, a state of near-emmetropic refractory is gradually reached. Emmetropization is still active in juvenile to adolescent stages, remaining emmetropia though a long period in axial length growth of eyes continuously. Even in the older ages, strong response to increased hyperopia by wearing a minus lens can be helpful 160, 165-167. In contrast, capability of emmetropization to adjust to a plus lens decreases quickly as aging. However, juvenile tree shrews wearing plus lenses cannot retard the elongation rate of eyes. In fact, elongation would continue, and eyes are still myopic even with lens 168. Without compensation of plus lenses, young, from juvenile to adolescent, tree shrews can recover quickly from induced myopia 154, suggesting that emmetropization may respond to myopic refractive state in an extended period in those animals 154, 169.

Recently, we have reviewed how myopia can be retarded by multifocal contact lenses 170. In fact, bright light can suppress myopia progression by activating dopamine D1 receptor 171. In addition, red light can promote progressive myopia and blue light may cause progressive hyperopia in chick model, and such inductions can be reversed to hyperopia and myopia if the red and blue lights are switched, suggesting that manipulation of chromaticity may produce beneficial effects to myopia 172.

Unfortunately, we still do not know why plus lenses do not slow down axial elongation or refractive compensation during aging of young animals. It is still unclear why normal eyes lack response to plus lenses while eyes with induced myopia can recover from axial elongation. It is probably in normal eyes, emmetropization may not retard axial elongation below threshold of a pre-programmed minimum. In the situation that eyes are elongated by minus lens wear, it is likely that sclera under remodeling of extracellular matrix has changed certain gene expression at mRNA and protein levels 173-175.

## Prospective for NIR Light Therapy

NIR light therapy is an effective method for treatment of various tissues such as eyes and brains without side effects. Researchers around the world should focus on delineating the action mechanism of NIR light for the therapeutic effects of various human diseases and provide scientific evidence to support it as a new major field of medicine. This therapy has potential to be a “miracle medicine” to cure many diseases in near future. Further work is needed for development of new NIR light equipment, establishment of various standard treatment protocols by NIR light therapy, and application of this therapy to various medical fields around the world.

## Conclusions

NIR light, or PBM, is a promising and powerful method to mediate biological functions via low power light wavelength from red to near-infrared regions. Eyes and neurons rely on cytochrome c oxidase to generate energy for metabolic process. NIR light can penetrate these tissues and assist recoveries of neurons in methanol intoxication, optic nerve trauma and neuropathy, retinal injuries and pigmentosa, and macular degeneration. NIR light can also help brains to recover from atherothrombotic stroke, brain injury, and neurodegeneration. No side effects have been observed from animals and humans. Therefore, NIR light could be a safe and effective method for a wide range of applications in ophthalmological and neurological fields in the near future.
